# Human Sulfatase 2 inhibits *in vivo *tumor growth of MDA-MB-231 human breast cancer xenografts

**DOI:** 10.1186/1471-2407-10-427

**Published:** 2010-08-13

**Authors:** Sarah M Peterson, Andrea Iskenderian, Lynette Cook, Alla Romashko, Kristen Tobin, Michael Jones, Angela Norton, Alicia Gómez-Yafal, Michael W Heartlein, Michael F Concino, Lucy Liaw, Paolo GV Martini

**Affiliations:** 1 Maine Medical Center Research Institute, 81 Research Drive, Scarborough, ME 04074, USA; 2 Shire Human Genetic Therapies Inc., 700 Main Street, Cambridge, MA 02139, USA

## Abstract

**Background:**

Extracellular human sulfatases modulate growth factor signaling by alteration of the heparin/heparan sulfate proteoglycan (HSPG) 6-*O*-sulfation state. HSPGs bind to numerous growth factor ligands including fibroblast growth factors (FGF), epidermal growth factors (EGF), and vascular endothelial growth factors (VEGF), and are critically important in the context of cancer cell growth, invasion, and metastasis. We hypothesized that sulfatase activity in the tumor microenvironment would regulate tumor growth *in vivo*.

**Methods:**

We established a model of stable expression of sulfatases in the human breast cancer cell line MDA-MB-231 and purified recombinant human Sulfatase 2 (rhSulf2) for exogenous administration. *In vitro *studies were performed to measure effects on breast cancer cell invasion and proliferation, and groups were statistically compared using Student's t-test. The effects of hSulf2 on tumor progression were tested using *in vivo *xenografts with two methods. First, MDA-MB-231 cells stably expressing hSulf1, hSulf2, or both hSulf1/hSulf2 were grown as xenografts and the resulting tumor growth and vascularization was compared to controls. Secondly, wild type MDA-MB-231 xenografts were treated by short-term intratumoral injection with rhSulf2 or vehicle during tumor growth. Ultrasound analysis was also used to complement caliper measurement to monitor tumor growth. *In vivo *studies were statistically analyzed using Student's t test.

**Results:**

*In vitro*, stable expression of hSulf2 or administration of rhSulf2 in breast cancer cells decreased cell proliferation and invasion, corresponding to an inhibition of ERK activation. Stable expression of the sulfatases in xenografts significantly suppressed tumor growth, with complete regression of tumors expressing both hSulf1 and hSulf2 and significantly smaller tumor volumes in groups expressing hSulf1 or hSulf2 compared to control xenografts. Despite significant suppression of tumor volume, sulfatases did not affect vascular density within the tumors. By contrast, transient exogenous treatment of MDA-MB-231 xenografts with rhSulf2 was not sufficient to inhibit or reverse tumor growth.

**Conclusion:**

These data indicate that *in vivo* progression of human breast cancer xenografts can be inhibited with sulfatase expression, and therapeutic effect requires constant delivery at the tumor site. Our results support a direct effect of sulfatases on tumor growth or invasion, rather than an effect in the stromal compartment.

## Background

Breast cancer is the leading cause of invasive cancer in women. The lifetime risk of breast cancer in women is estimated at 1 in 8 (13% of women) [1]. Factors that decrease cancer cell invasion and tumor growth have the potential for translation into novel therapeutic approaches for lessening breast cancer morbidity and mortality. Extracellular sulfatases appear to have a significant role in cancer biology. Heparan sulfate-like glycosaminoglycans, along with structural proteins, are important regulators at the cell surface-extracellular matrix (ECM) interface [2,3]. Human Sulfatase 1 (hSulf1) and Sulfatase 2 (hSulf2) are heparan sulfate 6-*O*-endosulfatases, a family of secreted enzymes that are either localized on the cell surface or released into the ECM. HSulf1 and hSulf2 cleave 6-*O*-sulfate moieties of heparan sulfate, thereby affecting composition and function of the glycosaminoglycans. Because glycosaminoglycans regulate cytokine signaling, heparan sulfate sulfation patterns and pattern-dependent cell signaling events rely on activity of sulfatase enzymes. HSPGs are key components of the ECM that are involved in tumor progression by regulation of growth factor signaling pathways. Most of the molecular events associated with tumor growth, neovascularization, and metastases are influenced by interactions between cells and their ECM. There is mounting evidence for the role of two secreted human extracellular sulfatases, hSulf1 and hSulf2, in modulating the growth factor signaling pathways needed for tumor angiogenesis and progression [4]. However, much of the data that exists on the role of hSulf1 and hSulf2 is context dependent to distinct cancer types and cellular environment [5-8].

HSulf1 and hSulf2 have overlapping yet distinct roles both in development and cancer progression. Mouse knockout models of Sulfatase 1 or Sulfatase 2 display a normal phenotype by nearly all criteria examined [9]. Combined knockout of Sulfatase 1 and 2 in mice, however, leads to ~50% neonatal lethality and ~80% postnatal lethality with prominent skeletal and renal developmental defects [9]. Cancer models also display evidence of context-specific sulfatase activity. HSulf1 expression is decreased in 82% of hepatocellular carcinoma (HCC) cell lines, leading to increased HSPG sulfation, enhanced FGF-mediated and hepatocyte growth factor (HGF)-mediated signaling, and increased cell growth [10]. Forced expression of hSulf1 decreases sulfation, inhibits growth factor signaling, and sensitizes HCC cells to chemotherapeutic apoptosis [10]. While previous study of overexpression of hSulf1 in the estrogen receptor negative MDA-MB-468 cell line has been shown to decrease tumor burden in athymic nude mice *in vivo*, few data are available regarding the therapeutic role of forced expression of hSulf2 on *in vivo *tumor burden.

We hypothesized that hSulf2 might have an effect in inhibiting cancer cell growth and invasion *in vitro *and *in vivo*. In order to further understand the role of hSulf2 in breast cancer, we chose the human breast cancer cell line MDA-MB-231, a well characterized and established model for human breast cancer growth. Although reports in the literature describe variable levels of sulfatase expression in MDA-MB-231 cells [6,11], we did not detect endogenous expression of hSulf1 or hSulf2 in our MDA-MB-231 population, using RT-PCR. We therefore chose the MDA-MB-231 cell line for gain-of-function studies by creating transfected pools that stably express human sulfatases. Given that hSulf1 has been established as an inhibitor of tumorigenesis [8], we used overexpression of hSulf1 as a positive control and also tested hSulf1 in conjunction with hSulf2. For delivery of rhSulf2, intratumoral injection was chosen as the initial approach for two reasons. The first was based on *in vivo *data about the anticipated effect of hSulf2 on the stromal component. The second was based on attempt to avoid first pass metabolism through the liver and deliver maximal protein to the tumor site.

*In vitro *studies provide evidence that expression or delivery of hSulf2 decreases proliferation and tumor cell invasion through the ECM. *In vivo *studies show that hSulf2 expression is effective in suppressing xenograft growth. In our system, this effect appears to be independent of an angiogenic effect, because tumor growth was significantly suppressed without dramatic changes in the angiogenic response to the xenograft. Further studies are needed to better characterize and validate the role of hSulf2 in inhibiting tumor growth and progression *in vivo*.

## Methods

### Materials

FGF2 and HB-EGF-2 were obtained from Sigma (St. Louis, MO). EGFR inhibitor PD153035 was obtained from EMD Biosciences (San Diego, CA). Total ERK and phospho-ERK antibodies were purchased from R&D Systems (Minneapolis, MN). Growth factor reduced Matrigel was obtained from BD Biosciences (San Jose, CA). CD31 (PECAM) antibody was obtained from BD Pharmingen. Ki-67 antibody was obtained from Ventana Corporation (Tucson, AZ).

### Recombinant protein production

An expression vector with full-length human Sulfatase 2 cDNA was transfected into HT1080 cells by electroporation. HT1080 cells were grown and maintained in a serum-free DMEM/F-12 based custom media (Invitrogen, Carlsbad, CA) at 37°C in a 5% CO_2 _incubator. Transfection with a neomycin resistance cassette allowed for the selection of stably transfected clones. Clones were expanded and cell supernatants examined for expressed hSulf2 protein. Conditioned medium was processed by copper binding followed by gel filtration. Selected gel filtration fractions were pooled and dialyzed against 20 mM sodium phosphate, 0.5 M NaCl, 10% glycerol, 0.5 mg/ml Pefabloc at pH = 7.5. RhSulf2 was visualized by Coomassie staining (GelCode Blue Stain Reagent, Pierce, Rockford, IL) of 8-16% tris-glycine SDS-PAGE gels (Invitrogen, Carlsbad, CA). Glycerol and Pefabloc were removed by dialysis into a final buffer of 500 mM NaCl, 20 mM NaPO_4 _pH = 7.

### Cell Culture

MDA-MB-231 (HTB-26) and MDA-MB-435 S (HTB-129) cell lines were obtained from ATCC. MDA-MB-231 cells stably expressing control vector, human Sulfatase 1 (hSulf1), human Sulfatase 2 (hSulf2), or a combination of human Sulfatase1 and 2 (hSulf1/hSulf2) were established. Specific clones of transfected hSulf1 and hSulf2 cells were selected based on the strength of respective hSulf protein and phenotypic validation *in vitro *of reduced cell proliferation and migration. MDA-MB-231 and MDA-MB-435 S cells for *in vitro *experiments were cultured in minimal essential media (MEM) supplemented with 10% FBS, 2 mM L-glutamine and 0.01 mg/ml insulin at 37°C, 5% CO_2_. For the invasion assay, cells were cultured in MEM supplemented with 5% charcoal-stripped FBS, 2 mM L-Glutamine and 0.01 mg/ml insulin 24 hours prior to assay. On the day of the assay, cells were suspended in migration media (serum-free basal media) and placed in the top well of invasion chambers (Chemicon ECM554). Chemoattractant (10% FBS) was placed in the lower chamber in migration media. Cells were allowed to invade for 24 hours at 37°C. Cells were harvested and invasion rate was determined according to manufacturer's protocol. For MTT assays, cells were seeded at 40,000 cells/cm^2 ^into 48-well plates in complete growth medium. After 24 h, cells were rinsed and treated with either rhSulf2 formulation buffer or with varying concentrations of rhSulf2. On days 1, 2 and 3, 5 mg/ml MTT was added to the cells for 4 hours at 37°C. After the incubation the media was aspirated, DMSO was added and the OD at 570 nm was measured. Cell count and viability was assessed using a Cell sorter (Cedex, Innovatis-Roche, Germany). For tumor xenograft implantation, MDA-MB-231 cell populations expressing human sulfatases were cultured in MEM supplemented with 10% FBS, 1% non-essential amino acids, and 0.5% gentamicin.

### RT-PCR

Primers used for hSulf1 were S1fw 5' ACGGGGGAGCTGGAGAATACTTAC 3'/S1rev 5' GCCACTTCTGCCCCGGTTGTTCAC 3', for hSulf2 there were 2 sets of primers S2fw 5' CCGCCCAGCCCCGAAACC 3'/S2rev 5' CTCCCGCAACAGCCACACCTT 3' as well as S2fw 5' CTCCGTTTTCCTTTGTGAGC 3'/S2rev 5' GAATTTGCAACTGGCTTCCT 3' and for β-actin 5' AGAAAATCTGGCACCACACC 3'/5' CTCCTTAATGTCACGCACGA 3' was used. One Step RT-PCR kit (Invitrogen, Carlsbad, CA) was used according to manufacturer's instruction for sulfatase determination in cells.

### Stable transfectant tumor xenograft production

All animal studies were performed in accordance with established protocols approved by the Maine Medical Center Institutional Animal Care and Use Committee. Three groups of stably transfected MDA-MB-231 cells were prepared for *in vivo *tumor xenograft growth in comparison with control vector-transfected cells: cells expressing both hSulf1 and hSulf2, only hSulf1, or only hSulf2. Thirty-two litter-matched female NCr homozygous *nu/nu *mice (Taconic) at nine weeks of age were chosen as xenograft hosts. These were randomized by cage into four groups of eight mice each. The first group was injected with the control vector-transfected cells, the second group was injected with cells expressing both hSulf1 and hSulf2, the third group was injected with cells expressing only hSulf1, and the fourth group was injected with cells expressing only hSulf2. Injections were placed subcutaneously into the left flank and contained 5 million MDA-MB-231 cells suspended in 200 μl of PBS. Measurements were obtained by caliper length and width measurements at 2-3 day intervals for the duration of the experiment. Tumor volume was calculated from the pi-based ellipsoid volume formula π/6*length*width*height [12], assuming ellipsoid shape with equal width and height. The average value and standard deviation are based on calculated tumor volumes from the eight mice in each group. Tumor xenografts recovered from mice were fixed in 4% paraformaldehyde and embedded in paraffin. Tissue sections were stained with Masson's trichrome staining for visualization of collagen, CD31 (PECAM) staining for visualization of tumor vasculature, or Ki-67 staining for mitotic index. Average mitotic index quantification was obtained by counting 5 high-power fields per tumor using ImageJ software [13].

### Tumor xenograft production for exogenous therapy with purified recombinant human Sulfatase 2 (rhSulf2)

For production of tumor xenografts for the exogenous treatment arm of the study, 200 μl of a 1:1 Matrigel:buffer suspension containing 8.5 million MDA-MB-231 cells was subcutaneously injected into the left flank of 32 female litter-matched nude mice. Four additional female litter-matched nude mice were injected with 200 μl of the Matrigel:buffer suspension only, and four remaining female litter-matched nude mice were left un-manipulated as experimental controls. Following a 48 hour window to allow establishment of tumor xenografts in mice injected with tumor cells, intratumoral injections of rhSulf2 were administered to the 16 mice randomized to the treatment group. Each injection contained 0.1 mg rhSulf2 based on an estimated 5 mg/kg dose in an average 20 g nude mouse. An equal volume of control buffer vehicle was administered intratumorally to the 16 mice in the treatment control group.

### High resolution ultrasound imaging of tumor xenografts

Mice were anesthetized by inhaled isoflurane prior to ultrasound scanning with a VisualSonics Vevo 770 high-resolution imaging system. Tumors were initially scanned free hand to establish approximate tumor size and morphology. Images for tumor reconstruction were acquired by 3D motor stage in alignment with the long axis of the tumor. After scanning, images were processed on a high-definition monitor for obtaining volumetric measurements. Successive tracings of tumor contour were taken which enabled the computer software to generate a 3D image and volume for each tumor. For comparison to external caliper measurements, × axis (length) was assigned to the longest visible dimension of the tumor. From there, y axis (width) was assigned to 90 perpendicular axis, and Z axis (height) assigned to tumor depth. The pi-based ellipsoid equation π/6*length*width*height has previously been validated as the best equation for estimating subcutaneous tumor size in athymic nude mice [12]. However, tracking of tumor size by estimation of standard caliper measurements *in vivo *involves the assumption of width equaling depth, given that only two dimensions can be measured *in vivo*. Here we report methodology for making these measurements more precise by non-invasively capturing depth component through high resolution *in vivo *ultrasound imaging.

### Statistical Analysis

Statistical analyses were performed using Student's t test, with a significant difference determined as p < 0.05. Where appropriate, data are represented as means ± SD.

## Results

### HSulf2 expression in MDA-MB-231 cells

**Figure 1:**
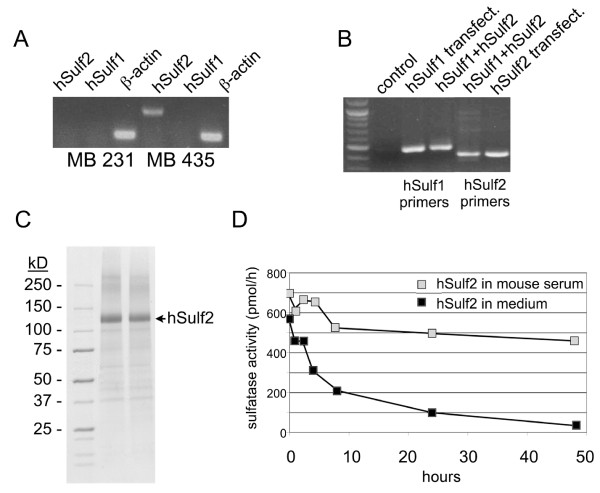
**Sulfatase expression in a human breast cancer model and analysis of recombinant human Sulfatase 2**. A) Human breast cancer cell lines MDA-MB-231 (MB 231) and MDA-MB-435 S (MB 435) were tested for expression of Sulfatase1 (hSulf1), Sulfatase2 (hSulf2), or β-actin by RT-PCR. B) RT-PCR was used to detect hSulf1, hSulf2 or both sulfatase transcripts in MDA-MB-231 after stable transfection with human sulfatase-containing plasmids. C) Coomassie staining of SDS-PAGE gel shows a prominent band at 120 KDa corresponding to purified rhSulf2 loaded in both lanes. D) Purified hSulf2 protein was incubated at 250 nM in medium or mouse serum, and activity assayed over time.

Most of the recent data have been focused on the effect of hSulf1 in different tumor cell lines. HSulf1 over-expression in cells has been reported to inhibit tumor formation *in vitro *and *in vivo*. We were interested in understanding the role of the related protein hSulf2 in breast cancer. While MDA-MB-435 S cells expressing Sulfatase 2 can represent a valid model to study the effect of endogenous Sulfatase 2 in breast cancer, we have chosen MDA-MB-231 cells as a model for our study because they have undetectable levels of endogenously produced human Sulfatase 1 or Sulfatase 2 as analyzed by RT-PCR (Fig. 1A). MDA-MB-435 S cells in comparison have detectable endogenous expression of Sulfatase 2 as shown in Fig. 1A. In addition, MDA-MB-231 is an estrogen-independent cell line that does not require exogenously added estrogen for xenograft growth. This was an advantage in our study to focus specifically on changes in sulfatases, because estrogen has diverse effects on tumor phenotype and angiogenesis [14,15]. Thus, MDA-MB-231 cells were used for gain-of-function studies to understand their roles in breast cancer cell phenotype. We generated MDA-MB-231 cells that stably express human Sulfatase 2 or Sulfatase 1, or a combination of both sulfatases. HSulf1 stably transfected MDA-MB-231 cells were used as a positive control based on previous publication on the effect of expression of hSulf1 in breast cancer cells [8]. Following transfection with sulfatase constructs, expression was confirmed by RT-PCR with specific primers (Fig. 1B). In addition, we were interested in understanding the effect of recombinant hSulf2 as a possible therapeutic for breast cancer. We have expressed and purified recombinant hSulf2 (rhSulf2) in the HT1080 human cell line. Coomassie staining shows a prominent band at about 120 KDa, corresponding to rhSulf2 loaded in both lanes (Fig. 1C). After purification, rhSulf2 was tested for activity based on its ability to convert 4-methylumbelliferyl sulfate (4-MUS) into fluorescent 4-methylumbelliferone as previously described [16]. The activity of hSulf2 was relatively stable over the course of 2 days if diluted in mouse serum, which had a protective effect on rhSulf2, and extended its biological activity over time compared to rhSulf2 stored in cell medium at 37°C (Fig. 1D). These results suggest that rhSulf2 could potentially be used for *in vivo *studies due to its long lasting activity in serum.

### Modulation of MDA-MB-231 cell invasion and proliferation by hSulf2

**Figure 2:**
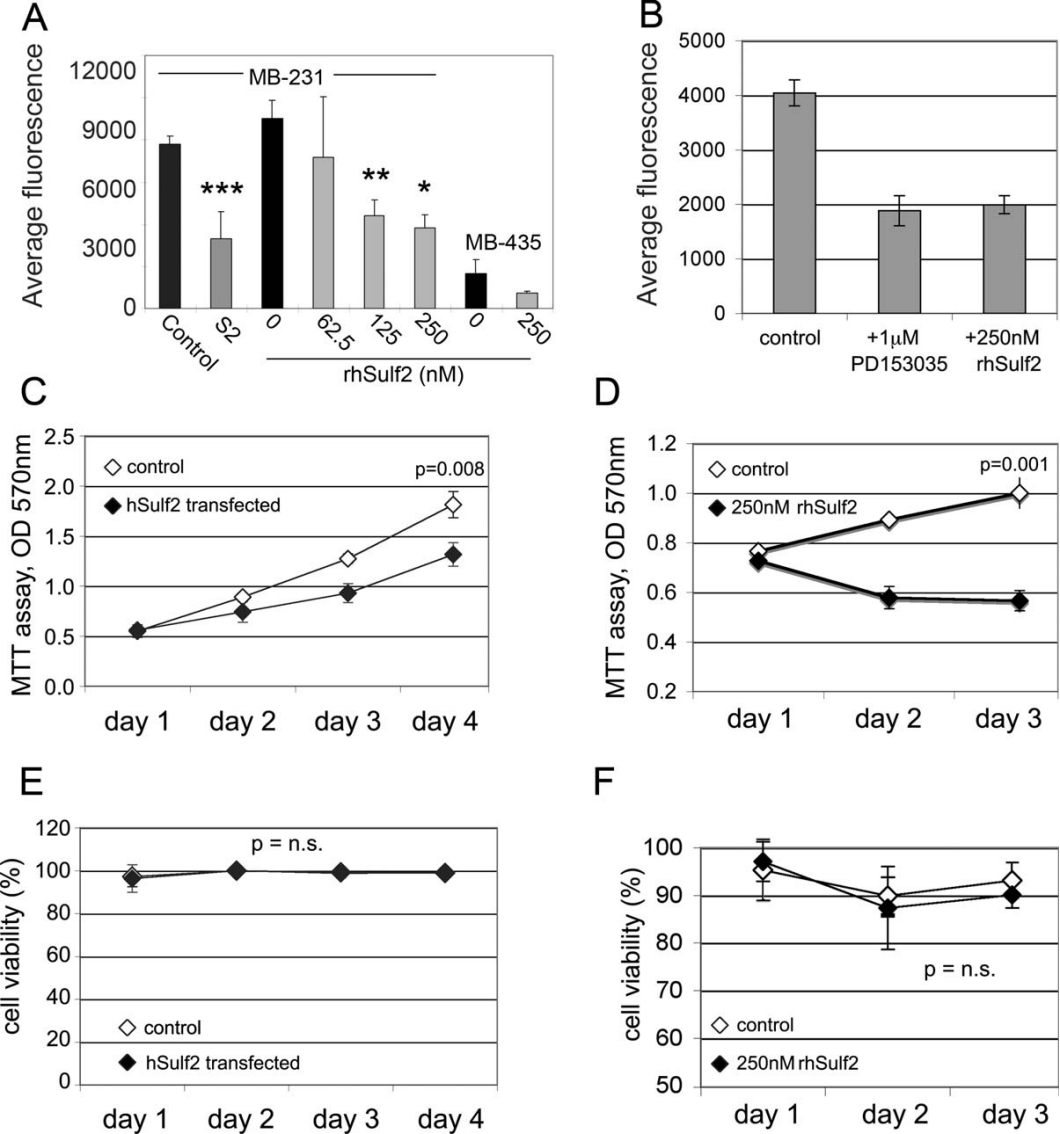
**Sulfatase 2 decreases breast cancer cell invasion and proliferation**. A) MDA-MB-231 (MB-231) and MDA-MB-435 S (MB-435) cell invasion was assayed with treatment of recombinant hSulf2 protein compared to buffer treatment (0). Stably hSulf2 transfected cells (S2) were also compared to control vector transfected cells for invasive ability * = p < 0.002; ** = p < 0.001; *** = p < 0.04. B) Invasion assays were performed with MDA-MB-231 cells in the presence of the EGFR kinase inhibitor PD153035 or rhSulf2. C) For the proliferation assay, cells were plated and collected over 4 days to test mitochondrial reductase metabolic activity using the MTT assay. Decreased MTT conversion in hSulf2 transfected cells corresponded to non-proliferating cells during this period. D) Recombinant human Sulfatase 2 protein (rhSulf2) was added to MDA-MB-231 cells at 250 nM, and cells assayed over 3 days using the MTT assay. E) Cell viability is expressed as percent of live cells and corresponds to data represented in panel C. F) Corresponding cell viability data for panel D; n.s. = not significant.

The effect of sulfatases in inhibiting cell invasion has been investigated by over-expression of hSulf1 in different cancer cells [4,8]. But, to date, recombinant human Sulfatase 2 effect on breast cancer cells has not been tested *in vitro *or *in vivo*. We treated MDA-MB-231 cells with different concentrations of rhSulf2 and we observed a dose-dependent decrease in invasion through an ECM barrier with the highest effect at 125 nM or 250 nM rhSulf2 compared to buffer control (Fig. 2A). As a positive control we tested MDA-MB-231 cells that were stably transfected with either hSulf1 or hSulf2. HSulf1 was shown by Narita *et al*. [8] to decrease invasion of MDA-MB-468 breast cancer cells. HSulf1 and hSulf2 stably transfected MDA-MB-231 cells confirmed previous data from MDA-MB-468 cells and our results obtained by treating the cells with rhSulf2 also showed inhibition of cell invasion. It is interesting to note that when we looked at the invasion rate of MDA-MB-435 S cells, expressing endogenous Sulfatase 2, we have observed a lower degree of invasion compared to MDA-MB-231 cells. The lower invasion rate of MDA-MB-435 S was further suppressed by adding 250 nM rhSuf2, suggesting that hSulf2 may play a role in regulating metastases in breast cancer. Furthermore, rhSulf2 inhibited invasion at a comparable rate to the known EGFR-dependent kinase inhibitor PD 153035 [17] (Fig. 2B). Given the role of growth factors in promoting cell proliferation and knowing that sulfatases might influence the interaction of the growth factor receptors with signaling molecules, we tested the ability of hSulf2 to inhibit cell proliferation *in vitro*. We measured cellular metabolic activity (Fig. 2C) of MDA-MB-231 cells stably expressing hSulf2 and found decreased metabolic activity and reduced proliferation without change in cell viability (Fig. 2E) compared to control cells. Interestingly, treatment of MDA-MB-231 cells with rhSulf2 inhibited the growth of cells compared to controls (Fig. 2D) without affecting cell viability (Fig. 2F), similar to the effect of endogenously expressed hSulf2. This suggests that hSulf2 is probably interfering with growth factor stimulation of the cells, as previously demonstrated.

### HSulf2 inhibits growth factor-induced ERK activation

**Figure 3:**
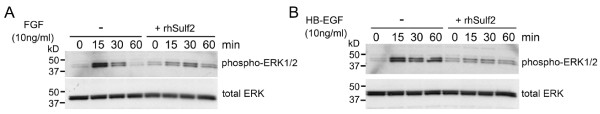
**Recombinant human Sulfatase 2 inhibits growth factor-induced ERK activation**. MDA-MB-231 cells were treated with 250 nM rhSulf2 for 24 h prior to FGF (A) or HB-EGF (B) stimulation. Cell lysates were collected before growth factor addition (0 min), or 15, 30, and 60 minutes after addition for analysis of phospho-ERK1/2 and total ERK. The two phosphorylated p44 and p42 species of ERK are detected using phospho-ERK1/2 antibodies.

It is speculated that sulfatases contribute to heparan sulfate remodeling on the tumor cell surface impairing growth factor signaling in the cells. It has been shown that hSulf1 in particular inhibits ERK1/2 phosphorylation through the FGF receptor. We wanted to determine the mechanism by which hSulf2 affects cell proliferation. MDA-MB-231 cells were treated with 250 nM rhSulf2 for 24 hours prior to stimulation with FGF2 or HB-EGF. Cell lysates were collected before (0 min) or 15, 30, and 60 minutes following growth factor addition (Fig. 3). Treatment with rhSulf2 led to suppressed growth factor-induced phosphorylation of ERK1/2. Our data correlate with previous studies using hSulf1 that demonstrate inhibition of FGF signaling and growth both *in vitro *and *in vivo *in human cancer cells [4,7].

### Stable expression of sulfatases in MDA-MB-231 cells inhibits *in vivo *tumor xenograft growth

**Figure 4:**
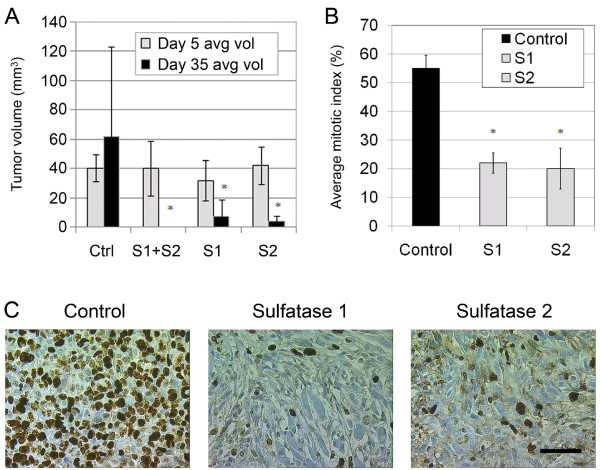
**Stable expression of sulfatases in MDA-MB-231 cells inhibits *in vivo *tumor xenograft growth**. A) Average tumor volume at day 35 study endpoint for control vector transfected MDA-MB-231 cells compared with hSulf1 and hSulf2 transfected (S1+S2), hSulf1 (S1), and hSulf2 (S2) transfected cells. The average value and standard deviation are based on caliper measurements and calculated tumor volumes from the eight mice in each group *p < 0.03. B) Average mitotic index at day 35 study endpoint for hSulf1 (S1) and hSulf2 (S2) transfected tumors in comparison to control as determined by Ki-67 staining *p < 0.001. C) Representative fields used for quantification of mitotic index by Ki-67 staining. The scale bar represents 50 μm..

Expression of hSulf1 in human cancer cell xenograft *in vivo *inhibits tumor growth and progression [4,7]. No studies are currently available on the effect of hSulf2 on tumor xenograft *in vivo*. Therefore we tested two *in vivo *xenograft protocols to determine the effects of hSulf2 on tumor growth. As a positive control we used MDA-MB-231 hSulf1 expressing cells. The two sulfatases were also co-expressed to test for a cooperative effect between hSulf1 and hSulf2. The stable transfectants were injected subcutaneously and monitored to compare sulfatase-expressing cells with empty vector control cells (Fig. 4). All groups formed tumors initially and there was no significant difference in average tumor volume at day 5. Tumor xenografts composed of MDA-MB-231 cells stably co-expressing hSulf1 and hSulf2 (S1 + S2) demonstrated complete regression (Fig. 4, p < 0.02), with no tumors remaining by day 35 after injection. Tumor xenograft groups expressing either hSulf1 or hSulf2 demonstrated partial regression with significant decreases in average tumor volume (p < 0.03 and p < 0.02 respectively). The fact that tumors co-expressing hSulf1 and hSulf2 formed tumors initially that later regressed suggests that a threshold of persistent sulfatase activity in the tumor microenvironment may be required for its anti-tumor activity. While MDA-MB-231 xenograft growth rate and size varies amongst studies reported in the literature, our tumor xenografts were in range with those reported previously [18,19]. Tumor xenograft size variability and variable growth rate may be due to MDA-MB-231 cell line heterogeneity (as opposed to clonal expansion of a single cell population) as well as variation in strain of immunocompromised mouse host. NCr homozygous nude mice have intact innate immunity, which includes anti-tumor natural killer cell activity.

**Figure 5:**
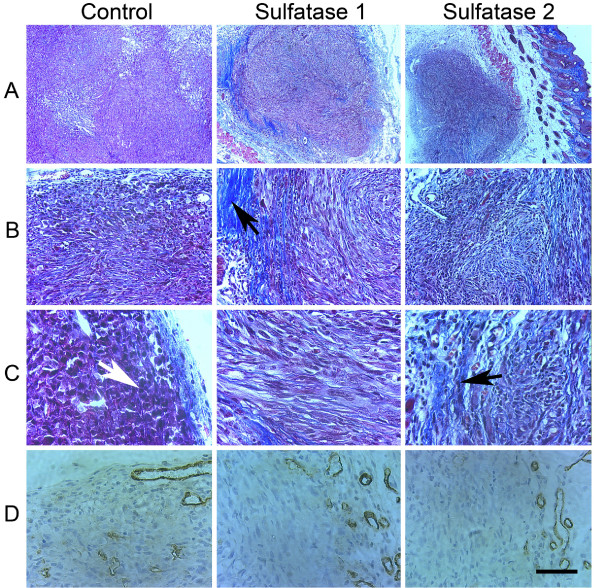
**Histopathological features of tumor xenografts from MDA-MB-231 cells transfected with control vector, Sulfatase 1 or Sulfatase 2**. A-C) Masson's trichrome staining of tumor xenografts. D) CD31 (PECAM) staining of tumor xenografts. The scale bar represents 400 μm for row A, 100 μm for row B, and 50 μm for rows C and D. Control group tumors exhibited higher cellular density and prominent mitotic activity (white arrow in row C), whereas the Sulfatase 1 and Sulfatase 2 group tumors contained more connective tissue and collagen (black arrows in rows B and C). For the tumors present, vessel density quantification of CD31 (PECAM) staining showed no significant differences between groups. Analogous data for the transfected tumors co-expressing Sulfatase 1 and 2 are not available due to tumor regression.

Control group tumors exhibited a significantly higher average mitotic index as demonstrated by Ki-67 staining (Fig. 4B, p < 0.001 and 4C). Control group tumors also displayed higher cellular density (white arrow in Fig. 5). Correlating with recent studies of sulfatase expression in myeloma cells [4], our experimental tumor xenograft groups expressing sulfatases show comparatively increased ECM deposition and collagen (black arrows in Fig. 5). Because of the possibility that tumor-derived sulfatases could impact both tumor and stromal cells, the vascular response in the tumors was evaluated by CD31 (PECAM) antibody staining of endothelial cells, followed by quantification of vessel area. There was no significant difference in average microvascular density among tumors in the control, hSulf1 or hSulf2 transfected groups (data not shown). This finding correlates with the observation in myeloma tumor xenografts that inhibition of tumor xenograft formation was not dependent on changes in microvessel density [4]. Although *in vitro *cell viability was not regulated by sulfatases, the *in vivo *phenotype shows tumor regression following the establishment of a small tumor, particularly with the combined expression of hSulf1 and hSulf2. Thus, hSulf2 expression in tumor cells leads to a phenotype consistent with cell intrinsic and localized effect to suppress growth of tumor cells. Dai et al. postulate that restricted sulfatase activity within the tumor microenvironment *in vivo *is due to heparan sulfate remodeling on the tumor cell surface rather than the surrounding ECM [4]. Experimental support for this model was the assembly of an FGF2 tertiary signaling complex in the tumor stroma but not on the surface of tumor cells [4]. This is further supported by the fact that a restricted local effect of Sulf1 has been shown by Ai, et al. who demonstrated that quail Sulf1 only alters the heparan sulfate on the cell expressing the enzyme as opposed to adjacent cells that lack Sulf1 expression [20].

### Exogenous short term intratumoral administration of rhSulf2 is not sufficient for inhibition of tumor xenograft growth

**Figure 6:**
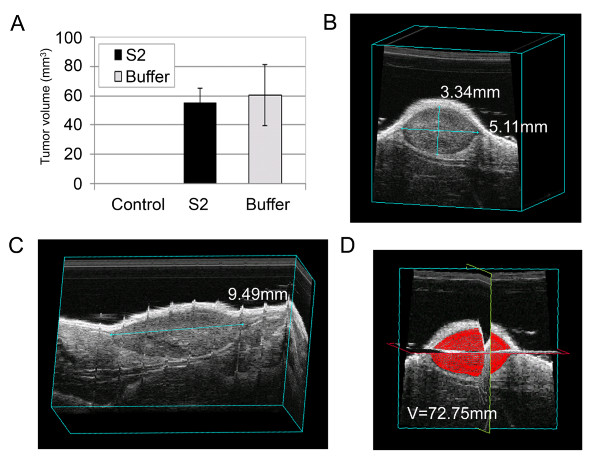
**Exogenous treatment with short course therapy of purified hSulf-2 insufficient for tumor xenograft inhibition**. Athymic nude mice were randomized into two experimental groups (n = 16 each) and injected with MDA-MB-231 cells. On day 2 after injection, intratumoral injections were started with rhSulf2 or equivalent volume of vehicle. No significant difference was noted in average tumor xenograft volume after short course intratumoral injections of purified rhSulf2. Average volume as estimated by high resolution 3D ultrasound scanning of tumors at day 9 (A). Two methods for estimation of tumor size are shown in panels B-D. In the first method, width and height measurements (B) in combination with length (C) can be used in ellipsoid volume calculations as described above. Alternatively, tumor tracings in multiple frames (C) can be used to recreate a 3-dimensional tumor reconstruction and estimated volume (E). Given the irregular nature of these tumors, this second method was employed for capturing aberrancies from true ellipsoid shape. In this case, ultrasound-estimated volume is ~73 mm^3 ^compared to an equation-calculated volume of ~85 mm^3^. This smaller volume is not surprising considering that caliper measurements include the overlying subcutaneous tissue whereas intra-ultrasound caliper measurements measure only the tumor itself.

As a second method to test sulfatase activity in tumor growth, we formed xenografts with MDA-MB-231 cells (non-transfected), and treated growing tumors by intratumoral injection of rhSulf2 or buffer vehicle. RhSulf2 injections were started 2 days after cell injection, and continued for 7 consecutive days. There was no significant difference in average tumor volume between the rhSulf2 treated mice or the buffer vehicle treated mice at day 9 after tumor cell injection (Fig. 6A). The differences observed with this protocol versus the stable transfectants may be due to lack of persistent, tumor-derived sulfatase expression. As noted above, the source of the sulfatase and its ability to mediate functional enzymatic activity seems to require activity near tumor cell surfaces. This may explain why transfected tumor cells exhibited dramatic inhibition of tumor xenograft growth while it was not possible to mimic these results with exogenously administered rhSulf2 into the general tumor environment. In addition, the variability between individual tumor growth patterns in this experiment could also account for lack of significant decrease in tumor volumes after a short treatment period. Average tumor volume was additionally obtained by three-dimensional (3D) reconstruction of the tumor by high-resolution ultrasound scanning. As expected, control mice not injected with cells or buffer-matrigel suspension did not develop spontaneous tumors. The control mice injected with buffer-matrigel suspension initially developed palpable nodules at the injection site due to the matrigel suspension that spontaneously regressed within 9 days (Fig. 6A). For simplicity, the control data point is based on the eight mice included in both control branches. These studies validate the use of ultrasound as a highly quantitative method to monitor 3D growth of tumors over time. This method may be particularly useful for the tracking of tumors growing internally on or within solid organs.

## Discussion and Conclusions

The results presented in this study suggest a potential role for hSulf2 in inhibiting cancer growth and metastases. We have demonstrated that cell invasion through an ECM-enriched matrigel is strongly inhibited when the cells are expressing hSulf2 or are in the presence of rhSulf2, and this effect is dose-dependent. The inhibitory effect of hSulf2 on MDA-MD-231 cells is comparable to the one observed with the classical EGFR-dependent kinase inhibitor PD153035. HSulf2 inhibits cell growth and proliferation of cells, and it does so by interfering with growth factor signaling.

Until recently, investigations into the action of hSulf1 and hSulf2 focused on growth factor modulation and angiogenesis. In the non-sulfatase expressing breast cancer cell line MDA-MB-468, forced expression of hSulf1 led to inhibition of angiogenesis and tumorigenesis in xenografts [8]. While there is consensus regarding the action of hSulf1 and hSulf2 in the desulfation of heparan sulfate, there is controversy over whether this effect is pro-angiogenic [6,21] or anti-angiogenic [4,6,8,10,21]. Specifically, studies in carcinoma cell lines producing hSulf2 demonstrate that MCF-7-derived hSulf2 decreases heparin sequestration of VEGF, FGF1 and selected chemokines to promote growth factor activity by prevention of binding and dissociation of complexes that are already bound [21]. Similarly, Sulf2 promotes angiogenesis in the chick chorioallantoic membrane assay [6]. In contrast, studies of other breast, pancreatic, renal, and hepatocellular carcinoma cell lines in which Sulf1 is down-regulated, suggest that endosulfatase activity inhibits angiogenesis. One potential explanation for this is that sulfatases may display additional effects beyond modulation of angiogenesis in specific contexts.

Interestingly, hSulf2 has recently been identified as a novel transcriptional target of the tumor suppressor gene p53 indicating that it may be a p53 downstream effector molecule [8]. This finding is particularly significant in the context of mutant p53 status in MDA-MB-231 and MDA-MB-468 cells and wild-type p53 status in MCF-7 breast cancer cell lines [22,23]. Knockdown of mutant p53 by RNA interference causes massive apoptosis in mutant p53 breast cancer cell lines but not in wild-type p53 breast cancer cell lines, indicating that mutant p53 may confer oncogenic potential as well as loss of tumor suppressor activity [23]. Knock-down of Sulf2 in MDA-MB-231 cells confers enhanced survival characterized by increased proliferation and anchorage-independent growth [11]. Our data are consistent with this work, because expression of hSulf2 had the opposite phenotype in decreasing tumor cell growth. Our findings extend the *in vitro *work to provide the first description of *in vivo *breast cancer xenograft growth inhibition by sulfatases.

Ai *et al*. [24] demonstrated that avian endosulfatases, Sulf1 and Sulf2, associate with the cell membrane and are enzymatically active on the cell surface to desulfate HSPG. Desulfation of HSPG by endosulfatases has been shown to impair growth factor signaling through FGF and EGF receptors [4,7]. In this study we have demonstrated that rhSulf2 decreases ERK1/2 phosphorylation as previously found for hSulf1 and hSulf2. Therefore, our data support decreased growth factor signaling as the mechanism of inhibition of tumor growth. Combinatorial therapy using conventional chemotherapy and hSulf2 could provide an alternative and more effective option, although further study is needed to determine whether hSulf2 could potentially be an effective exogenous therapy for specific subtypes of breast adenocarcinoma.

While data from sulfatase-transfected tumors are promising, a detailed *in vivo *study is required to reproduce these results with exogenous administration of rhSulf2. Although short term intratumoral administration of rhSulf2 was not efficient in inhibiting tumor growth, our data suggest that constant production of hSulf2 is required for a theraupeutic effect. In addition, secretion of hSulf2 in stable transfectants from early stages of xenograft growth may have modified the tumor cells or microenvironment. Although we performed a pilot study to administer rhSulf 2 by intravenous tail vein injection, this approach was limited by the high osmolarity of buffer vehicle in a tail vein injection distal enough to allow for adequate proximal intravenous access for subsequent daily delivery (data not shown). In addition, the concentrations of purified proteins were not sufficiently high to allow for injection of small enough volumes. While this could potentially be circumvented by jugular venous cannulation for central line access, the concentration of protein accumulated at the tumor site is predicted to be much lower than compared to intratumoral injection. Development of strategies for therapeutic exogenous administration of sulfatases requires in depth pharmacokinetic analysis for determination of volume of distribution, rate of metabolism, and dose-response curves in different contexts. Given the complexity of cellular events during malignant transformation and tumor progression in addition to the context-dependent nature of sulfatase activity, it is not surprising that the delivery method is critical to tumor response. Potential complicating factors to be considered in the future include active concentrations in the tumor microenvironment, the timing and duration of sulfatase administration, and a requirement for tumor cell expression for intrinsic growth effect. However, our studies provide proof of principle that sulfatase within a human breast cancer xenograft can lead to tumor suppression and indeed regression of established tumors.

## Abbreviations

HSPG: heparin/heparan sulfate proteoglycan; FGF: fibroblast growth factor; EGF: epidermal growth factor; VEGF: vascular endothelial growth factor; Sulf2: Sulfatase 2; hSulf2: human Sulfatase 2; rhSulf2: recombinant human Sulfatase 2; Sulf1: Sulfatase 1; hSulf1: human Sulfatase 1; MB 231: MDA-MB-231 breast cancer cell line; ERK: extracellular signal-related kinases; ECM: extracellular matrix; HCC: hepatocellular carcinoma; HGF: hepatocyte growth factor; RT-PCR: reverse transcription polymerase chain reaction; HB-EGF: heparin-binding epidermal growth factor-like growth factor; EGFR: epidermal growth factor receptor; MEM: minimal essential media; FBS: fetal bovine serum; MTT: 3-(4,5-Dimethylthiazol-2-yl)-2,5-diphenyltetrazolium bromide; DMSO: dimethyl sulfoxide; OD: optical density; PBS: phosphate buffered saline; 4-MUS: 4-methylumbelliferyl sulfate; 3D: three-dimensional; RNA: ribonucleic acid.

## Competing interests

Financial disclosure: Project funding was provided by Shire Pharmaceuticals. AI, LC, AR, AN, AG-Y, MWH, MFC, and PGVM are current employees of Shire Human Genetic Therapies Inc.

## Authors' contributions

PGVM conceived the idea of this study. PGVM, AI, LC, AN, AR, MFC, MWH and AGY designed and/or performed the *in vitro *experiments. SMP, KT, and LL designed and performed the *in vivo *experiments. SMP and LL drafted the initial manuscript, prepared the figures, an edited subsequent revisions. MJ performed the histopathology analysis. PGVM critically revised the manuscript. All authors read and approved the final manuscript.

## Pre-publication history

The pre-publication history for this paper can be accessed here:

http://www.biomedcentral.com/1471-2407/10/427/prepub
